# Retrospective analysis of OCT parameters after intravitreal anti-VEGF inhibitors in neovascular AMD patients in a real-world setting

**DOI:** 10.1007/s10792-022-02383-6

**Published:** 2022-07-04

**Authors:** Jan Niklas Lüke, Hamdi Alquoqa, Ahmad Alsamman, Bayan Aljabary, F. Schaub, L. M. Heindl

**Affiliations:** 1 Department of Ophthalmology, Faculty of Medicine and University Hospital, University of Cologne, Kerpener Str. 62, 50937 Cologne, Germany; 2 KRH Klinikum Nordstadt, Hannover, Germany; 3 Augenärzte Mühlenquartier, Gifhorn, Germany; 4 Universitätsmedizin Rostock, Klinik und Poliklinik für Augenheilkunde, Rostock, Germany

**Keywords:** AMD, Age-related macular degeneration, Retrospective interventional study, Non-response, Anti-VEGF

## Abstract

**Purpose:**

The aim of the present study was to evaluate changes of best corrected visual acuity (BCVA), retinal nerve fiber layer thickness (RNFL), total macular volume (TMV), intraocular pressure (IOP) and central retinal thickness (CRT) after intravitreal injection of ranibizumab, bevacizumab and aflibercept in patients with neovascular age-related macular degeneration (nAMD) in a clinical real world setting.

**Methods:**

In a retrospective clinical study design, 120 patients (80 women and 40 men) were analyzed after being diagnosed with nAMD within 8 years (2010–2018). Every patient received at least 6 anti-VEGF injections in a Pro-Re-Nata or Treat-and-Extend regimen. OCT parameters (RNFL, TMV, CRT) and visual acuity (BCVA) were assessed at first diagnosis, at treatment day and during the course.

**Results:**

Intraretinal fluid was reduced significantly in a magnitude of 88–64 µm (CRT) and 0.75–0.55 mm^3^ (TMV). Apart from a significant reduction immediately after the therapy start (post-3 injections) with ranibizumab (− 1.4 µm, *p* = 0.03), RNFL thickness remained constant. A slight improvement in visual acuity of 0.06 logMAR could initially be observed. If further injections were required, only stabilization was achieved compared to baseline visual acuity.

**Conclusion:**

The changes of OCT parameters CRT, TMV, and RNFL as well as the stabilization of functional results (BCVA) as illustrated in this study comparing effects of different anti-VEGF-agents provide evidence for the transferability of former results to a clinical real-world setting.

## Introduction

In Central Europe, before glaucoma and diabetic retinopathy, age-related macular degeneration (AMD) is the major cause of irreversible loss of vision with a prevalence of more than 14% among all blind people [1]. By fundus examination, AMD can be clinically divided into early, intermediate and two late forms, exudative and dry AMD. A recently published meta-analysis showed that the visual acuity with the best possible correction (best corrected visual acuity; BCVA) of over 80-year-olds exudative AMD has improved significantly since 2006 [2]. This decrease is attributed to a healthier lifestyle and the introduction of anti-vascular endothelial growth factor (VEGF) therapy [2]. Typically, the response to anti-VEGF therapy can be monitored via BCVA and different optical coherence tomography (OCT) parameters such as central retinal thickness (CRT) or total macular volume (TMV) reflecting the extent of edema. In different clinical studies, the average reduction in CRT alternated between 96 and 216 µm [3–5].

### Anti-VEGF substances

Ranibizumab (Lucentis, Genentech, Inc., South San Francisco, USA) is a recombinant, humanized monoclonal antibody Fab fragment with a molecular mass of 48 kDa against VEGF, which is administered intravitreally with 0.5 mg per 0.05 ml injection. In 2007, ranibizumab was the first monoclonal antibody to be approved by the European Medicines Agency based on the multicenter, double-blind phase III studies ANCHOR and MARINA [6, 7]. Bevacizumab (Avastin, Genentech, Inc., South San Francisco, USA) is a humanized monoclonal antibody to VEGF with a molecular mass of 149 kDa, which is approved for the treatment of colorectal cancer, but is used off-label due to its lower price (EMA) [8]. It is administered with 1.25 mg per 0.05 ml intravitreally. Related to the BCVA, no significant differences between both agents could be found [9].

Aflibercept (Eylea/VEGF-TRAPR1R2, Regeneron Pharmaceuticals and Bayer AG, Berlin) is a recombinant fusion protein containing Ig domains of VEGFR-1 and -2 as well as the Fc fragment human IgG1. It binds VEGF-A, PlGF and VEGFB and is said to have a higher affinity and better bioavailability due to its structure [10]. Aflibercept is administered intravitreally with 2 mg per 0.05 ml injection and was approved by the European Medicines Commission in 2012 based on the VIEW 1/2 studies [11]. This study could not figure out any significant differences in preservation of BCVA compared to ranibizumab.

The response to anti-VEGF-therapy seems to depend on several factors like age, lesion characteristics, lesion duration, baseline visual acuity (VA) and the presence of particular genotype risk alleles [12]. Based on morphological characteristics and the functional improvement (BCVA), the response to therapy can be defined as poor (between 0 and < 25% reduction in CRT and/or persistence of subretinal fluid (SRF), intraretinal fluid (IRF), intraretinal cystoid fluid (IRC), and/or appearance of new IRC, IRF and SRF, change in VA of 0–4 letters), partial and good (absence of SRF, IRF, IRC or a reduction of CRT > 75% of the baseline values, improvement in VA > 5 letters). Non-responders show unchanging or increasing CRT, SRF, IRF and a change > − 5 letters from the baseline [12]. In cases of poor- or non-response, a switch of therapy-agents can be taken into account. Several studies describe evidence for a successful therapy switch [13–15].

Clinical experience has shown a mismatch between study conditions and real-world-data illustrating the treatment success of the anti-VEGF therapy in neovascular AMD. Due to the high morbidity and mortality of a very old patient population, adequate therapy is not always possible. The aim of this work was to investigate the effect of anti-VEGF injections in neovascular AMD reflecting real conditions of clinical care in Germany using OCT parameters CRT, TMV, RNFLT, intraocular pressure and visual acuity comparing ranibizumab, aflibercept and bevacizumab.

## Methods

### Cohort of patients

In this retrospective designed interventional study, eyes with neovascular AMD which received anti-VEGF therapy in the period from January 6, 2010, to November 6, 2018, were screened for eligibility.

Inclusion criteria were diagnosed neovascular AMD, treated with at least 6 injections of anti-VEGF (ranibizumab, bevacizumab or aflibercept) in an ophthalmologic medical care center in Braunschweig (Augenklinik & Augenarztpraxis Schlosscarrée MVZ Dr. Kamouna, Braunschweig, Germany) availability of sufficient pre- and postinjection clinical assessments including OCT, intraocular pressure (IOP) and BCVA data. A diagnosis of neovascular AMD was based on fundus examination, fluorescence angiography and OCT evaluation.

Patients with other potential OCT parameters influencing side diagnoses like glaucoma, ocular hypertension, myopia magna, state after vitrectomy, retinal laser photocoagulation or diabetic retinopathy were excluded. 25% of all patients underwent cataract surgery during anti-VEGF therapy which were not excluded.

Furthermore, eyes that underwent a switch of anti-VEGF agent during the study period were excluded from the main analysis.

According to national medical regulations on retrospective single center clinical studies, the Ethics Committee of the University of Cologne stated that an approval was not required for this study. All tenets of the declaration of Helsinki have been regarded.

### Groups and injection regimens

According to their insurance status, patients received intravitreal injections of 0.5 mg ranibizumab, 1.25 mg bevacizumab or 0.5 mg aflibercept in 0.05 ml solution. Included therapy regimes are Pro-Re-Nata (PRN) and Treat-and-Extend (TAE).

In the PRN regimen, the patients underwent a check-up 4 weeks after the initial injection series with visual acuity, measurement of intraocular pressure and OCT. Sub- and intraretinal fluid, new or persistent macular bleeding, an increase in pigment epithelium elevation and progressive loss of vision were considered as signs of progression, in which a further triple series of injections was implemented. The disease progression was evaluated according to the recommendations of the DOG, BVA and Retinological Society. Eyes without disease activity received monthly controls.

In contrast to this, in the TAE regimen patients were treated in intervals of 2 weeks longer after the loading phase in case of no progression at the control check-up appointment following the loading dose. In case of disease activity, the next intravitreal injection was applied after 4 weeks. For eyes without disease activity, the interval for the next injection was extended by 2 weeks up to 12 weeks during follow-up. In case of new disease activity during follow-up, the interval to the next IVI was reduced by 2 weeks with a minimum of 4 weeks.

### Clinical outcome parameter: visual acuity and optical tomography parameters

For visual acuity testing, best corrected visual acuity (BCVA) was measured. Results were converted to the Logarithm of the Minimum Angle of Resolution (logMAR). BCVA was tested at first diagnosis, treatment day and after each third injection during the follow-up. Depending on the treatment regimen OCT examinations were performed each treatment day (TAE) or after 3 consecutive injections (PRN).

All OCT examinations were performed by Spectralis-SD-OCT (Heidelberg Engineering, Heidelberg), software version 6.8.1. OCT scans were reviewed for segmentation errors and corrected manually if present.

**Fig. 1:**
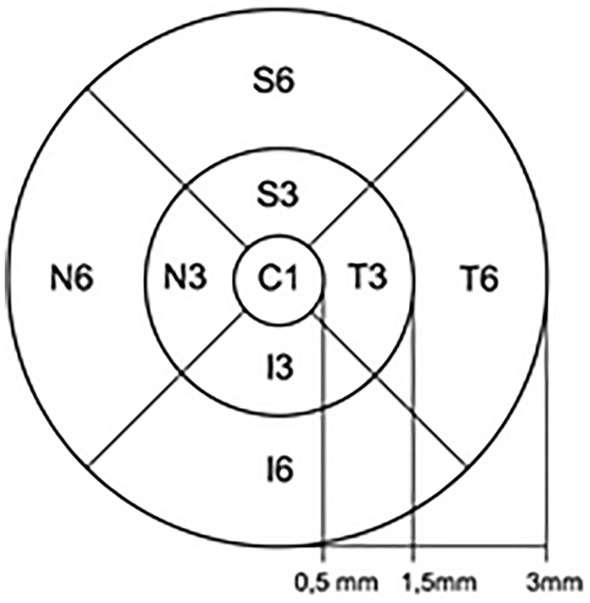
ETDRS grid. Early treatment diabetic retinopathy study design and baseline patient characteristics [16]

The quantitative evaluation of retinal layer thicknesses is based on the grid defined in the ETDR study, which is shown in Fig. 1 [16]. The central ring limiting C1 area is defined as the innermost 0.5 mm radius of the fovea centralis. The two outer rings with a radius of 1.5 and 3 mm are divided into 8 segments according to the temporal, nasal, superior and inferior. Central retinal thickness and total macular volume are established in the therapy monitoring of neovascular AMD for the evaluation of edema.

The central retinal thickness (CRT) is defined as the average thickness of the C1 segment in the ETDRS grid [16].

The total macular volume (TMV) is defined as the sum of all nine areas. The TMV is determined by default playing a clinically subordinate role. Standard values ​​are: 10.1 ± 0.6 mm^3^ [17]. In addition to the CRT, it serves as an additional marker for macular edema.

In this study, the RNFLT measurement was used as an additional parameter. In order to generate objective RNFLT values ​​from the available data set, this was determined in the outer temporal segment (T6, 1.5–3.0 mm) of the ETDRS grid, which is aligned with the temporal papilla margin. The segmentation was corrected manually.

### Statistical analysis

Data were collected and analyzed in Microsoft Excel (2016). For the confirmatory analysis of the measurement parameters, a paired t test (Graphpad Prism 5.0) was selected comparing the injection group (Post-X injections) with the respective baseline. *p* values < 0.05 were considered statistically significant. The analyzed parameters are given as mean change from baseline value and must be considered separately, since the baseline group, unlike in a prospective study, is different for each group analyzed (post-3–18 injections). However, the baseline groups are not independent of each other. Patients with a higher number of injections are always included in the previous groups.

## Results

### Patient groups and epidemiological data

120 patients (80 women, 40 men, mean age 78.5 ± 7.8 years of age) of whom 52 received ranibizumab, 41 bevacizumab and 27 aflibercept were eligible for outcome analysis. In the period from January 6, 2010, to November 6, 2018, the patients received at least 3 injections (mean number of injections 10 ± 4.2, range 3–18).

The exact periods of follow-up examinations were not recorded. In most cases, due to the multimorbidity and mean age of the cohort, follow-up examinations were not carried out accurately.

More than half of the patients (60.8%) underwent cataract surgery before or during anti-VEGF therapy. The patient characteristics are given in detail in Table 1. Table 2 displays the distribution in the injection groups.

**Table 1:** The main characteristics of patient selection

Group	Ranibizumab (*n* = 52)	Bevacizumab (*n* = 41)	Aflibercept (*n* = 27)	Total (*n* = 120)
Age—no. (%)				
60–69 years	5 (9.3)	3 (7.3)	1 (3.7)	9 (7.4)
70–79 years	28 (51.9)	18 (43.9)	10 (37.1)	56 (45.9)
80–89 years	18 (33.3)	16 (39.0)	15 (55.5)	49 (40.2)
≥ 90 years	3 (5.5)	4 (9.8)	1 (3.7)	8 (6.5)
Mean age	76.6 ± 8.7	79.7 ± 6.8	80.7 ± 6.1	78.5 ± 7.8
Sex—no. (%)				
Female	32 (61.5)	31 (75.6)	17 (63.0)	80 (66.7)
Male	20 (38.5)	10 (24.4)	10 (37.0)	40 (33.3)
DM type 2—no. (%)	9 (17.3)	8 (19.5)	5 (18.5)	22 (18.3)
Cataract surgery—no. (%)	27 (51.9)	23 (56.1)	23 (85.2)	73 (60.8)
Before treatment	15 (48.1)	7 (17.1)	21 (77.8)	43 (35.8)
During treatment	12 (23.1)	16 (39.0)	2 (7.4)	30 (25.0)
AMD subtype—no. (%)				
Occult	20 (38.5)	20 (48.8)	8 (29.6)	48 (40.0)
Classical	23 (44.2)	9 (22.0)	11 (40.7)	43 (35.8)
Hybrid	9 (17.3)	12 (29.3)	8 (29.6)	29 (24.2)
Average number of injections	10.8	9.3	9.3	10.0
CRT—µm	389 ± 92	396 ± 100	375 ± 100	389 ± 98
RNFLT—µm	54.5 ± 12.2	51.2 ± 9.9	48.7 ± 9.4	52.1 ± 11.8
TMV—mm3	9.0 ± 1.3	9.2 ± 1.7	9.0 ± 1.6	9.0 ± 1.4
IOP—mmHg	16.1 ± 3.0	15.5 ± 2.6	15.1 ± 3.5	15.7 ± 2.9
Visual acuity—logMAR	0.43 ± 0.23	0.55 ± 0.28	0.53 ± 0.32	0.49 ± 0.29

*AMD* age-related macular degeneration, *CRT* central retinal thickness, *SD* standard deviation, *TMV* total macular volume, *RNFLT* retinal nerve fiber layer thickness, *IOP* intraocular pressure, *logMAR* logarithm of the minimum angle of resolution

**Table 2:** The distribution in the individual groups in detail

Therapy	Ranibizumab	Bevacizumab	Aflibercept	Total
*Injection groups*				
Post-3	52	41	27	120
Post-6	52	36	27	115
Post-9	39	24	15	78
Post-12	24	15	8	47
Post-15	15	6	5	26
Post-18	6	5	2	13

### Best corrected visual acuity

**Fig. 2:**
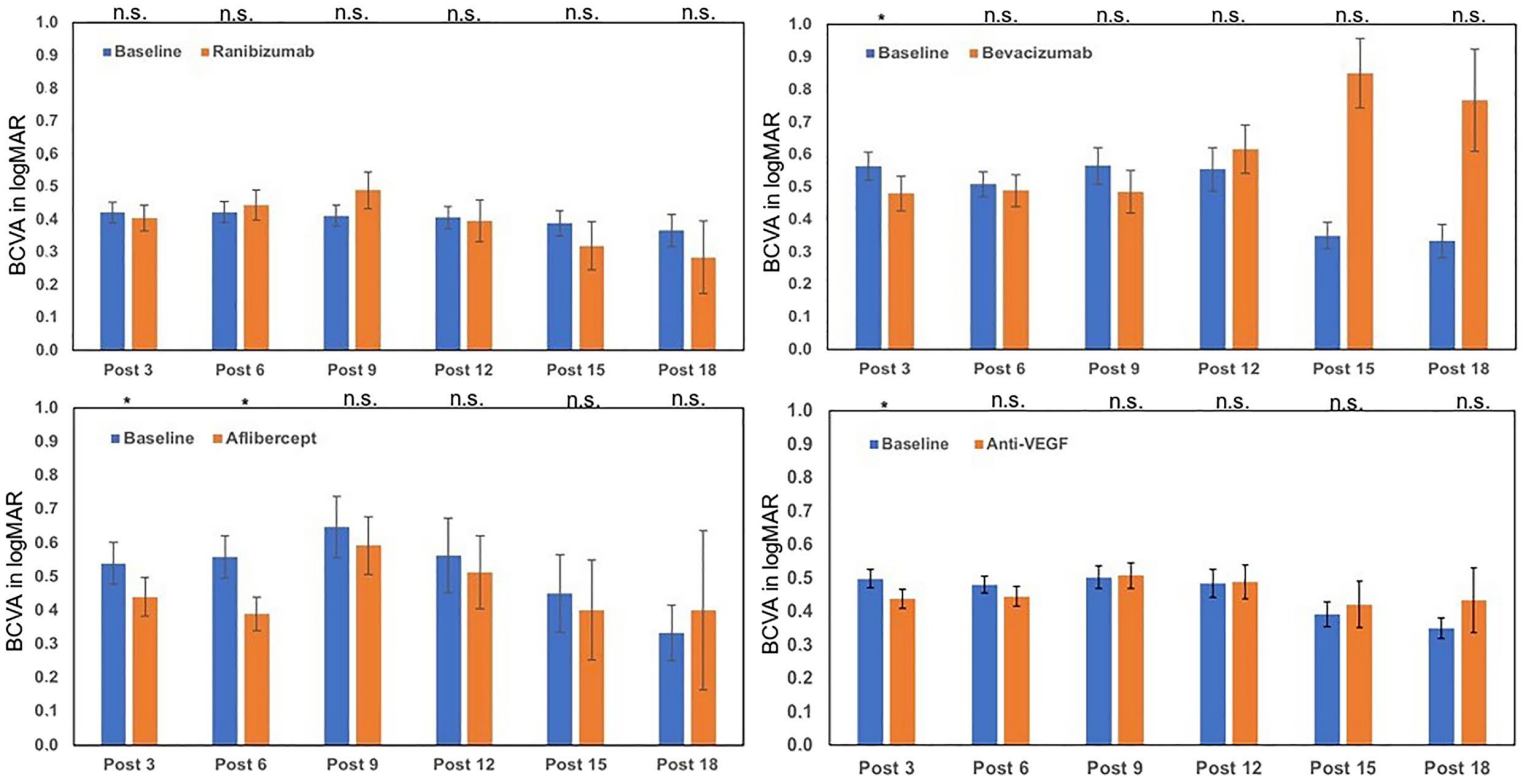
Alteration of visual acuity after 3–18 injections of ranibizumab, bevacizumab and aflibercept and all antibodies compared to baseline values; baseline values vary due to the different number of patients included in each group. *n.s.* not significant

The average best corrected visual acuity before treatment was low (0.49 logMAR). A statistically significant visual improvement could be found only in the post-3 group with a change of − 0.06 logMAR (*p* = 0.031). Injection groups post-3 to post-12 showed with an increasing number of injections stabilization of visual acuity, but no further improvement (post-12 VA difference = + 0.006, *p* = 0.93). The groups with the maximum of injections revealed a statistically insignificant worsened visual acuity (post-18 VA difference = + 0.08, *p* = 0.408). With a visual acuity of 0.42, there was better visual acuity in the ranibizumab group before treatment than in the bevacizumab and aflibercept group. The latter groups significantly benefit from anti-VEGF agents on the first three injections with a decrease of 0.09 logMAR for bevacizumab and 0.02 logMAR for aflibercept. In the aflibercept group, there is still a significant improvement even after 6 injections (VA difference = − 0.169; *p* = 0.021; Fig. 2).

### Central retinal thickness

**Fig. 3:**
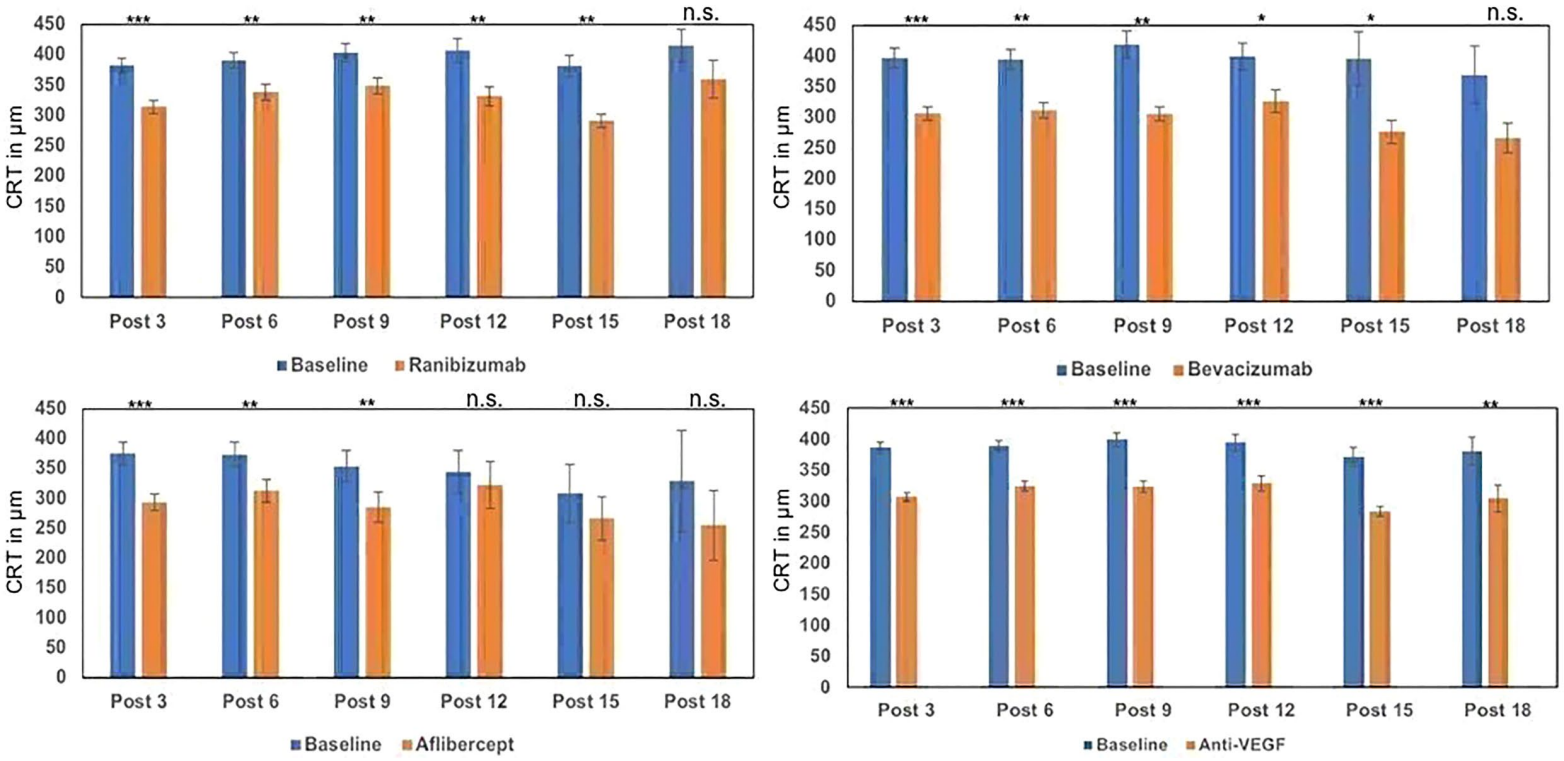
Alteration of CRT after 3–18 injections of ranibizumab, bevacizumab and aflibercept compared to baseline values. Baseline values vary due to the different number of patients included in each group. *n.s.* not significant

The average central retinal thickness (CRT) was 398 ± 98 µm before the start of therapy. Considering all different substances, the CRT reductions were statistically significant in a constant, clinically relevant order (between − 64 [post-6] and − 88 µm [post-15], *p* < 0.001 Fig. 3). All substances, illustrated individually, showed a significant reduction in macular edema in the course of the first 9 injections. Using ranibizumab and bevacizumab, up to 15 injections still showed a significant reduction. Due to the decreasing group sizes, the clinically relevant decreases in CRT that are similar in all groups are no longer significant with high injection numbers. The highest CRT reductions were observed with bevacizumab (post-15: − 119 µm, *p* = 0.04).

### Total macular volume

**Fig. 4:**
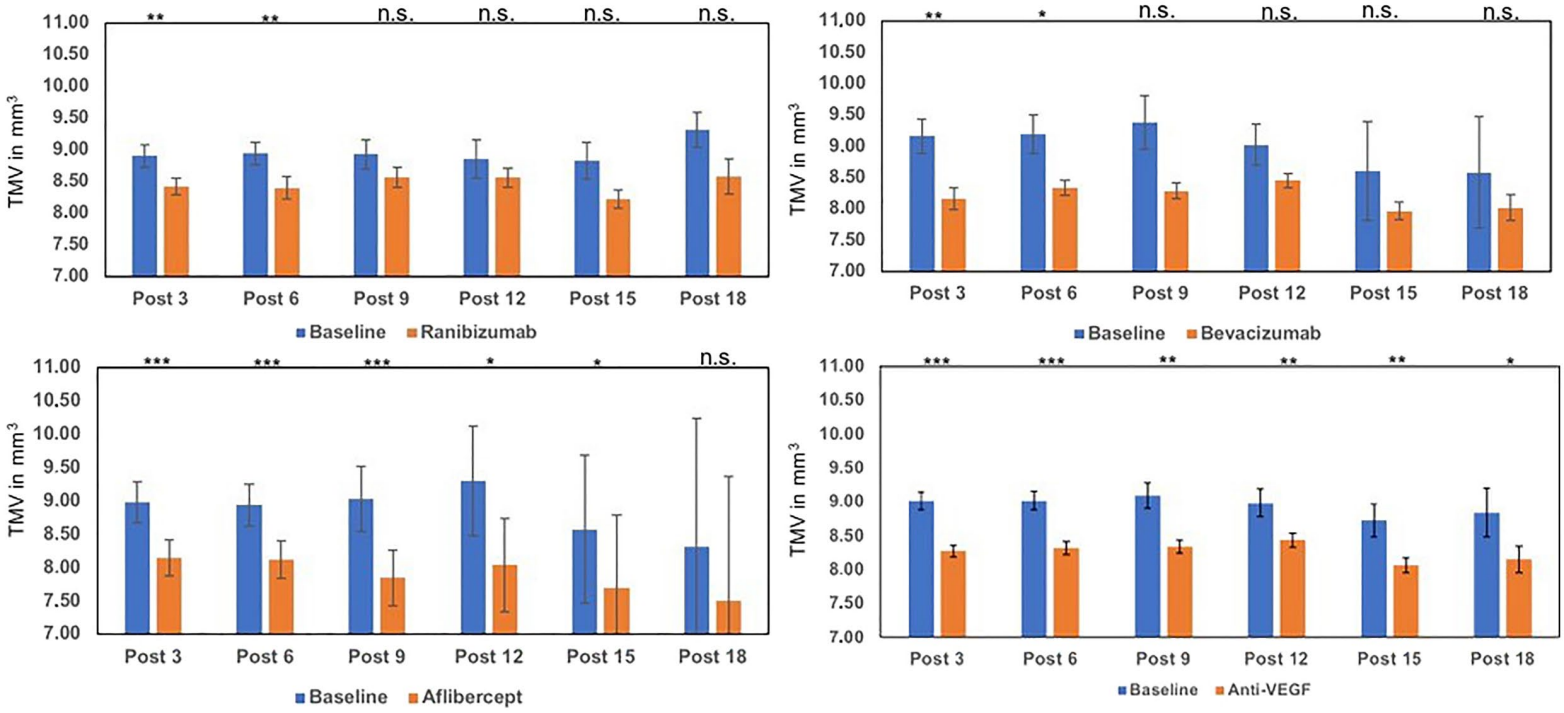
Alteration of TMV, after 3–18 injections of ranibizumab, bevacizumab and aflibercept and all antibodies compared to baseline values. Baseline values vary due to the different number of patients included in each group. *n.s.* not significant

Starting with an average total macular volume (TMV) of 9.0 mm^3^, a statistically significant reduction between − 0.55 mm^3^ (post-12, *p* < 0.01) and − 0.75 mm^3^ (post-9, *p* < 0.01) in all injection groups could be observed. For every single substance, there was a significant reduction in macular edema in course of the first 6 injections (− 0.7 mm^3^, *p* < 0.001). Similar to CRT, with constant reduction of edema, decrease of group size, and higher injection numbers TMV changes did not reveal further significant results. Quantitative differences of TMV changes between aflibercept (post-3: − 0.83 mm^3^), bevacizumab (post-3: − 1 mm^3^) and ranibizumab (post-3; − 0.49 mm^3^; Fig. 4) were not significant (bevacizumab vs. ranibizumab *p* = 0.24; aflibercept vs. ranibizumab *p* = 0.37*).*

### Retinal nerve fiber layer thickness

**Fig. 5:**
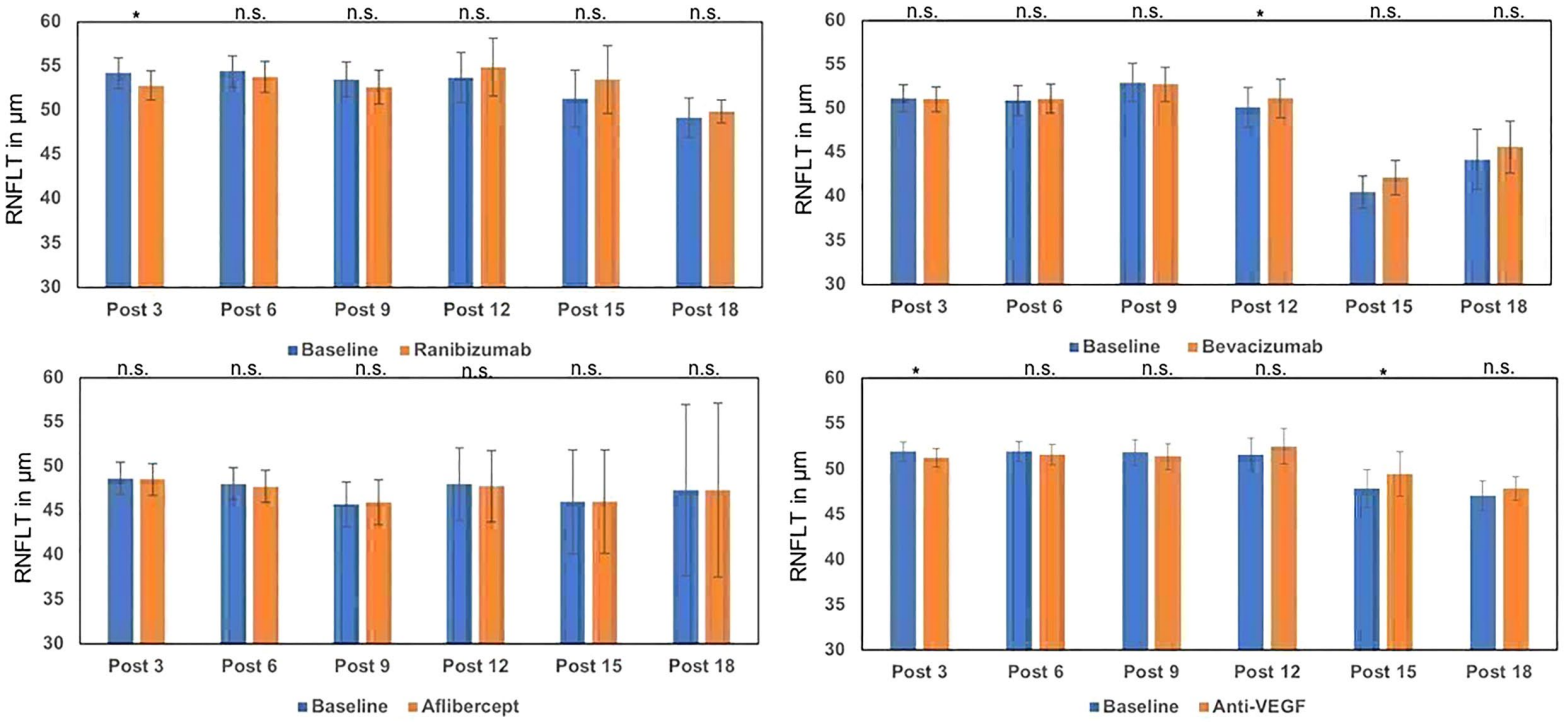
Alteration of RNFL, after 3–18 injections of ranibizumab, bevacizumab and aflibercept and all antibodies compared to baseline values. Baseline values vary due to the different number of patients included in each group. *n.s.* not significant

The average retinal nerve fiber layer thickness (RNFLT) was 51.9 µm including all eyes before the start of therapy. There were statistically significant changes in the post-3 (− 0.67 µm, *p* = 0.038) and post-15 injections groups (+ 1.62 µm, *p* = 0.03). RNFLT shows significant changes in two groups (ranibizumab post-3 [− 1.4 µm, *p* = 0.03] and bevacizumab post-12 [+ 1 µm, *p* = 0.05]). There were no significant changes in all other groups, respectively (Fig. 5).

## Discussion

In this study, the OCT parameters CRT and TMV as markers for the reduction of macular edema, RNFLT as a parameter for the integrity of the nerve fibers, visual acuity and eye pressure with different numbers of anti-VEGF injections from 120 patients with neovascular AMD in the clinical setting were examined.

CRT and TMV are commonly used for objective documentation of progress, whereas the BCVA represents the main outcome parameter of most studies.

Macular edema is significantly reduced after injection series up to 18 injections and in a constant size range of approximately 88–64 µm (CRT) or 0.75–0.55 mm^3^ (TMV). A slight improvement in the visual acuity of 0.06 logMAR can be observed initially. With further necessary injections, only stabilization is achieved compared to the initial visual acuity. This implies only a temporary improvement in visual acuity despite response to anti-VEGF therapy, corresponding to randomized controlled studies ANCHOR, IVAN, MARINA and CATT [6, 7, 18]. The last mentioned study could even figure out even a loss of BCVA in long-term follow-ups [19].

Similar to these studies, no differences between different anti-VEGF agents could be found [20].

However, the comparability of this study with the randomized controlled trials mentioned above is limited due to the lack of sharply defined treatment periods in this study.

### BCVA

As illustrated in the meta-analysis by Mehta et al. [21], numerous studies were published since the establishment of anti-VEGF injections as a clinical standard for nAMD showing less strong effects compared with the registration studies. The results of the German study population of the AURA study group have shown that in clinical real-world setting only a temporary, slight improvement in visual acuity is achieved after the loading phase [22]. Almost all examinations (> 98.7%) in the clinical setting have been irregular with longer intervals than 5 weeks. The importance of regular controls (4-weekly intervals) and the indication of regular OCT examinations has been pointed out by Ziemssen et al. More frequent sequences of controls and injections have a positive effect on improving and maintaining visual acuity during anti-VEGF therapy, which was demonstrated by a comparison of national differences in health systems with different effectiveness in therapy [23].

### CRT

As the most commonly used objective parameter, CRT was included as a criterion for progression in studies determining the intervals of therapy [3–5].

Furthermore, individual risk factors for treatment response were figured out [24]. High age and high CRT at baseline predicted high CRT reduction, whereas more injections, treatment with ranibizumab, and male sex predicted a low CRT reduction [24].

The magnitudes of CRT reduction among all studies differ a lot: In the SUSTAIN study population, an average CRT reduction of 101 µm after the loading phase was observed (baseline = 340.5 µm ± 113 µm), which remained constant in the further course of therapy [3]. In the PrONTO study, the mean CRT reduction after 12 months was 216 µm with a baseline of 394 µm [4]. In the EXCITE study, CRT was quantified 96–106 µm depending on the therapy regimen with baseline values ​​of 314–325 µm (± 85–119 µm) [5]. A subsumption of the results is limited due to the strong fluctuations in the CRT measurement between the listed studies and this one. Unlike Gabai et al. who pointed out a correlation between BCVA and CRT after 12 months in a real-world setting, the present study achieves a sufficient reduction in edema, whereas BCVA improvement was observed only after 3 injections [25]. Although the influence of CRT on the baseline BCVA is known, its significance during the course of therapy is discussed controversial [26].

A retrospective clinical study from Germany achieved similar results to the present study [27]. This study underlines that visual impairment and a purely quantitative determination of CRT (− 100 µm) are not sufficient as a criterion of progression, but that morphological criteria should always be taken into account [27].

### TMV

Total macular volume (TMV) is defined as the sum of all nine areas of the ETDRS grid. The reduction in TMV is significantly between − 0.55 and 0.75 mm^3^ for all anti-VEGF agents combined. TMV is more unconventional than CRT as a parameter for assessing therapeutic success, so that only a few studies are available. Ma et al. analyzed in a clinical setting 118 patients under the PRN regimen with bevacizumab [28]. The baseline TMV in this study is 10.2 ± 2.0 mm^3^. Unlike the present study, a strong reduction of 2.07 mm^3^ was observed after 9 month. The lower baseline value in our study (8.73–9.09 mm^2^) and the lower treatment adherence of our cohort due to the real-world study design can be discussed as possible reasons.

It serves as an additional marker for macular edema besides CRT. An advantage of TMV is the detection of CNV within a larger retinal area. However, this results in the disadvantage of a smaller discrimination range in which alterations take place. The differences between these studies underline that the TMV should be interpreted carefully as a progression parameter.

### IOP and RNFL

Increases in intraocular pressure during anti-VEGF therapy have been described in different studies [29]. A meta-analysis from 2015 determines 3.5–11.6% as the average IOP increase with a defined clinically relevant magnitude of > 21 mmHg and a minimum increase of 20% from the baseline value [30]. In our study, no significant changes in IOP could be observed. This meta-analysis included patients with glaucoma, whereas in our cohort these patients were excluded. A retrospective study showed that patients with pre-existing glaucoma experienced higher rates of elevated IOP when compared with patients without pre-existing glaucoma (33% vs. 3.1%, respectively; *p* < 0.001) [31]. The same study described a higher incidence of sustained elevation of IOP after bevacizumab. An explanation for this fact and elevated IOP levels in glaucoma patients after anti-VEGF injections in general could be that high molecular weight proteins, such as bevacizumab (150 kDa), which is approximately three times larger than ranibizumab may obstruct in the outflow and accumulate in the trabecular meshwork [30].

The small numbers of cases in the bevacizumab groups post-15 and post-18 with 6 and 5 eyes showing a significantly reduced baseline RNFLT limits the validity. The reduction of RNFLT (− 1.4 µm) in post-3 ranibizumab patients (*n* = 52) can be emphasized and discussed more in detail.

In meta-analysis of 2016 including 288 eyes, treated with different agents in a Pro-Re-Nata regime, no significant changes of RNFL were found in total [32]. In a subgroup analysis carried out in the same study, dividing the 288 eyes in subgroups with different numbers of injections [≥ 10 injections (*n* = 70) and < 10 injections (*n* = 218)], a significant decrease (MD = − 0.250, 95% CI − 0.441 to − 0.058, *p* = 0.011) was pointed out, similar to our observations in the post-3 group of ranibizumab. However, no long-term effects on RNFL with increasing numbers of injections of ranibizumab were neither observed in this study nor in several prospective designed studies using ranibizumab (*n* = 20–30) [33, 34]. Because of the elevated susceptibility for RNFL damage in glaucoma patients, these patients were excluded in our study. In regard to our findings, further prospective, well-designed, longitudinal examinations are needed to investigate the exact behavior of RNFL changes after ranibizumab injections.

### Limitations

As a retrospective study, the present study is limited in many respects. The number of patients decreases in groups of higher injection numbers, which is related to the low availability of patient data with long-term anti-VEGF therapy. As a result, systematic bias occurs at higher injection numbers: Patients in whom no therapeutic success can be achieved are underrepresented in these groups, because no therapy adherence is guaranteed under these circumstances. Furthermore, multimorbid and elderly patients, who represented the majority in our cohort, are less likely to be on long-term therapy. Similarly, patients in groups with higher injection numbers are mostly those with better baseline factors and favorable treatment outcome. The small number of patients in high injection numbers generally limits the significance of these results. Furthermore, it has to be pointed out that the number of injections correlates only slightly with the duration of therapy. However, this is urgently needed for an assessment of this chronic disease. The influence of the irregular therapy intervals is thus indirectly included in the overall outcome of the study. Statements about therapy intervals and therapy regimens were not considered in this study.

## Conclusion

The changes of OCT parameters CRT, TMV, and RNFL as well as the stabilization of the BCVA as illustrated in this study comparing effects of different anti-VEGF agents provide evidence for the transferability of former results to a clinical real-world setting.
